# FER promotes cell migration via regulating JNK activity

**DOI:** 10.1111/cpr.12656

**Published:** 2019-07-01

**Authors:** Ping Li, Zhiwei Ma, Yun Yu, Xingjie Hu, Yanfeng Zhou, Haiyun Song

**Affiliations:** ^1^ School of Public Health Shanghai Jiao Tong University School of Medicine Shanghai China; ^2^ Shanghai Institute of Nutrition and Health, Shanghai Institutes for Biological Sciences University of Chinese Academy of Sciences, Chinese Academy of Sciences Shanghai China; ^3^ School of Public Health Guangzhou Medical University Guangdong China

**Keywords:** cancer metastasis, cell migration, Fer/FER, JNK signalling

## Abstract

**Objectives:**

Cell migration has a key role in cancer metastasis, which contributes to drug resistance and tumour recurrence. Better understanding of the mechanisms involved in this process will potentially reveal new drug targets for cancer therapy. Fer is a non‐receptor protein tyrosine kinase aberrantly expressed in various human cancers, whereas its role in tumour progression remains elusive.

**Materials and Methods:**

Transgenic flies and epigenetic analysis were employed to investigate the role of *Drosophila* Fer (FER) in cell migration and underlying mechanisms. Co‐immunoprecipitation assay was used to monitor the interaction between FER and *Drosophila* JNK (Bsk). The conservation of Fer in regulating JNK signalling was explored in mammalian cancer and non‐cancer cells.

**Results:**

Overexpression of FER triggered cell migration and activated JNK signalling in the *Drosophila* wing disc. Upregulation and downregulation in the basal activity of Bsk exacerbated and eliminated FER‐mediated migration, respectively. In addition, loss of FER blocked signal transduction of the JNK pathway. Specifically, FER interacted with and promoted the activity of Bsk, which required both the kinase domain and the C‐terminal of Bsk. Lastly, Fer regulated JNK activities in mammalian cells.

**Conclusions:**

Our study reveals FER as a positive regulator of JNK‐mediated cell migration and suggests its potential role as a therapeutic target for cancer metastasis.

## 1 INTRODUCTION

Cell migration is a critical characteristic of cancer progression. This process facilitates cancer cells moving towards distant sites from the primary tumour and induces drug resistance.^1^, ^2^ Consequently, cancer metastasis contributes to >90% of the cancer mortality.^3^ Therefore, identifying specific genes involved in cancer cell migration and elucidating their underlying mechanisms hold great promise in the development of cancer therapies.

Cell migration is vital not only in pathological but also in physiological processes and is tightly regulated by various signalling pathways.^4^, ^5^ Among these pathways, the c‐Jun N‐terminal kinase (JNK) pathway plays crucial roles in both conditions.^4^, ^6^, ^7^, ^8^ In *Drosophila*, for example, JNK signalling is required for cell invasive migration induced by the Ret activation or Sin3a suppression.^9^, ^10^ Besides, during metamorphosis, JNK signalling promotes the eversion, spreading and fusion of imaginal epithelia.^11^

JNK, as an evolutionarily conserved mitogen‐activated protein kinase (MAPK), is activated in response to a variety of intrinsic and environmental stresses.^12^ The activation of JNK signalling is mediated by a core triple kinase cascade, consisting of JNK kinase kinases (JNKKKs, such as dTAK1 and Slpr), JNK kinases (JNKKs, Hep/MKK4) and JNK (Bsk in *Drosophila*).^13^

*Drosophila*, a classic model organism for developmental biological research, has recently been used to investigate the cancer‐related processes.^14^ Indeed, many cellular regulating systems and signalling pathways involved in the occurrence and development of cancer are evolutionarily conserved between human and flies.^15^, ^16^ This conservation enables *Drosophila* to model most hallmarks of mammalian cancer. Besides, a diversity of cancer models has been established in *Drosophila*.^17^, ^18^

Fer is a non‐receptor protein tyrosine kinase expressed ubiquitously.^19^ Early studies in Fer mainly focus on its role in regulating cell adhesion. Recent studies show that Fer is also implicated in numerous cellular processes such as cell cycle, cell proliferation, and migration.^19^ In addition, several lines of evidence support a role of Fer in cancer promotion. Firstly, it has been reported that Fer is aberrantly upregulated or activated in various cancers such as breast, prostate, hepatic and lung cancers.^20^, ^21^, ^22^, ^23^ Secondly, the high expression of Fer is significantly correlated with poor prognosis in renal cell carcinoma.^24^ Besides, a recent research reports that Fer overexpression activates NF‐κB pathway and confers quinacrine resistance to several cancer cell lines.^25^ Nevertheless, due to the complexity of cancer development and the limitations of our understanding of Fer, the molecular mechanism by which Fer promotes tumour progression remains elusive.

In this report, we investigate the function of FER, the *Drosophila* homolog of Fer, in a *Drosophila* model of cancer metastasis. Our results demonstrate that FER promotes cell migration in the wing disc, and the JNK signalling pathway mediates FER‐induced cell migration. Elevation or reduction in the activity of JNK signalling via genetic manipulations strengthens or suppresses FER‐induced cell migration, respectively. Besides, our work also reveals that FER is a positive regulator of the JNK signalling pathway and acts through Bsk. FER interacts with and phosphorylates Bsk, which require both the kinase domain and the C‐terminal of Bsk. Lastly, our data show that the regulatory role of FER in JNK signalling is conserved in mammalian cells.

## 2 MATERIALS AND METHODS

### 2.1 Fly strains and antibodies

All stocks were raised on standard *Drosophila* media at 25°C. The following stocks were used in this study: *ptc‐Gal4/cyo*, *en‐Gal4/cyo*, *GMR‐Gal4/TM6b*, *UAS‐GFP/cyo*, *UAS‐GFP/TM6b*, *UAS‐FER* (FlyORF: F001720), *FER^X42^* (Bloomington: 9361), *FER^KK^* (VDRC: 107266), *FER^GD^* (VDRC: 36053), *UAS‐mCherry‐IR/cyo* (gift from Professor Yanshan Fang), *UAS‐egr/cyo*, *UAS‐dTRAF2‐IR/TM6b*, *UAS‐dTAK1‐IR/TM6b*, *UAS‐hep‐IR/TM6b*, *UAS‐bsk‐IR/TM6b*, *UAS‐scrib‐IR/cyo*, *puc^E69^/TM6b* (gifts from Professor Lei Xue). Experiments with *UAS‐FER* were crossed at 25°C. Two days after egg laying, the F1 generations were shifted to 29°C.

The following antibodies were used: mouse anti‐MMP1 (3A6B4, 1:100) and rat anti‐E‐cad (DCAD2‐S, 1:100) were from DSHB. Rabbit anti‐p‐JNK (9251S, 1:200 for immunostaining; 1:1000 for Western blot) were from Cell Signaling Technology. Rabbit anti‐Arm (sc‐28653, 1:100), rabbit anti‐HA (sc805, 1:1000), rabbit anti‐Myc (sc789, 1:1000) and goat anti‐rabbit IgG‐HRP (sc‐2030, 1:3000) were from Santa Cruz Biotechnology. Goat anti‐mouse Alexa Fluor 594 (A11012, 1:500), goat anti‐rat Alexa Fluor 594 (A11007, 1:500) and goat anti‐rabbit Alexa Fluor 594 (A11005, 1:500) were from Invitrogen.

### 2.2 Immunostaining

Staining was carried out according to standard protocol.^26^ Briefly, third‐instar larva wing discs were fixed with 4% formaldehyde in PBS‐T for 20 minutes and blocked with 5% bovine serum albumin in PBS‐T for 30 minutes. Discs were then incubated with primary antibodies at 4°C overnight followed by incubating with secondary antibodies at room temperature for 2 hours. Mounted discs were analysed with a Zeiss LSM 880 confocal microscope and a Nikon DS‐Ri1 fluorescence microscope. Images were processed with Zeiss Zen and Adobe Photoshop software.

### 2.3 Co‐immunoprecipitation

Co‐immunoprecipitation was performed as previously described.^27^ Briefly, the transfected cells were lysed with lysis buffer at 4°C for 30 minutes. Insoluble fractions were removed, and supernatants were incubated with anti‐Myc agarose conjugates at 4°C for 4 hours. Then, the beads were washed with washing buffer and dissolved with SDS loading buffer.

### 2.4 X‐gal staining

Third‐instar wing discs were dissected in PBS and fixed with 1% glutaraldehyde in PBS‐T for 20 minutes. The samples were then stained for β‐galactosidase as previously described.^27^

### 2.5 Quantitative RT‐PCR

Total RNA from T24 cells was isolated using TRIzol reagent (15596018, Ambion) according to the manufacturer's instructions. Complementary DNA (cDNA) was reverse transcribed using the reverse transcriptase enzyme kit (FSQ‐101, TOYOBO). The housekeeping gene GAPDH was used as the internal control to normalize the mRNA levels. Quantitative PCR (qPCR) was done with an ABI StepOne Real‐Time PCR System (Applied Biosystems, Foster City, CA) using SYBR Green PCR Master Mix (QPK‐201, TOYOBO). Relative expression levels were calculated using the comparative CT method.^28^

## 3 RESULTS

### 3.1 FER promotes cell migration in the wing disc

**Figure 1:**
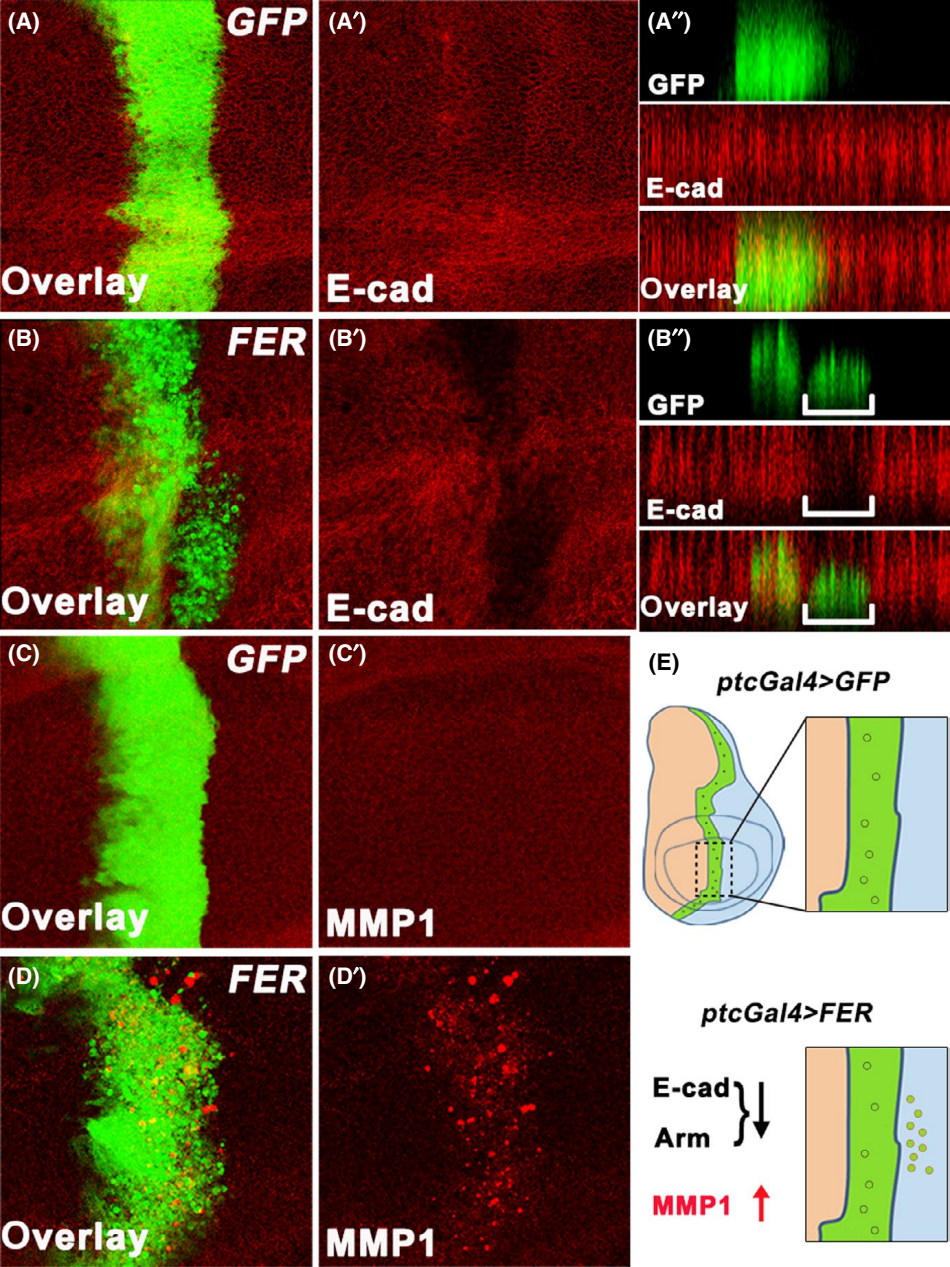
Overexpression of *FER* induces cell migration. A‐D, Immunostaining of E‐cad (A and B) and MMP1 (C and D) in the wing discs expressing *ptc‐Gal4 UAS‐GFP*/*+*; *UAS‐GFP*/*+* (A and C), *ptc‐Gal4 UAS‐GFP*/+; *UAS‐FER*/+ (B and D). (A’’ and B’’) Z stack images of the wing discs. White brackets highlight the reduced E‐cad signals in the migrated area. (E) A scheme for FER‐induced cell migration in the *Drosophila* wing disc

It has been reported that Fer is able to modulate cell migration in numerous cell lines.^29^, ^30^ To explore the precise function of Fer in an intact organism, we employed the *Drosophila* wing disc epithelium paradigm, a well‐established system to study cell migratory behaviour in *Drosophila*.^31^ We ectopically expressed *Drosophila Fer* (*FER*) at the A/P compartment boundary of the wing disc by *patched‐GAL4* (*ptc‐Gal4*). Compared to the control, many FER‐expressing cells migrated from the A/P compartment boundary to the posterior part of the wing disc (Figure 1A‐B, and Figure S1). In addition, the x‐z imaging of these discs showed that FER‐expressing cells aberrantly localized basally and moved to the posterior compartment, away from the main body of *ptc‐*Gal4* > FER* cells (Figure 1A’’‐B’’). Thus, the overexpression of FER leads to cell migration in the *Drosophila* wing disc.

Cell migration is initiated with the epithelial‐mesenchymal transition (EMT) and accompanies with increased expression of matrix metalloproteases (MMPs).^32^ Therefore, we further investigated the effects of ectopic FER expression on EMT and MMPs expression. During EMT, cell‐cell adhesion is disrupted, which can be indicated by aberrant levels of E‐cadherin (E‐cad) and β‐catenin/Armadillo (Arm).^10^, ^33^ Immunostaining assays showed that overexpression of FER significantly induced downregulation of both E‐cad and Arm (Figure 1A‐1B, and Figure S2), suggesting that FER promoted the destabilization of adherens junctions. MMPs are a conserved family of proteases that regulate cell‐cell and cell‐extracellular matrix (ECM) contacts. There are two MMPs in *Drosophila*, MMP1 and MMP2. MMP1 is reported to involve in *Drosophila* cell invasive migration and tumour metastasis.^34^, ^35^ Consistently, we found that overexpression of FER markedly improved the expression of MMP1 (Figure 1C‐D, and Figure S3). Together, these results confirm that overexpression of FER promotes cell migration (Figure 1E).

### 3.2 FER activates JNK signalling in the wing discs

**Figure 2:**
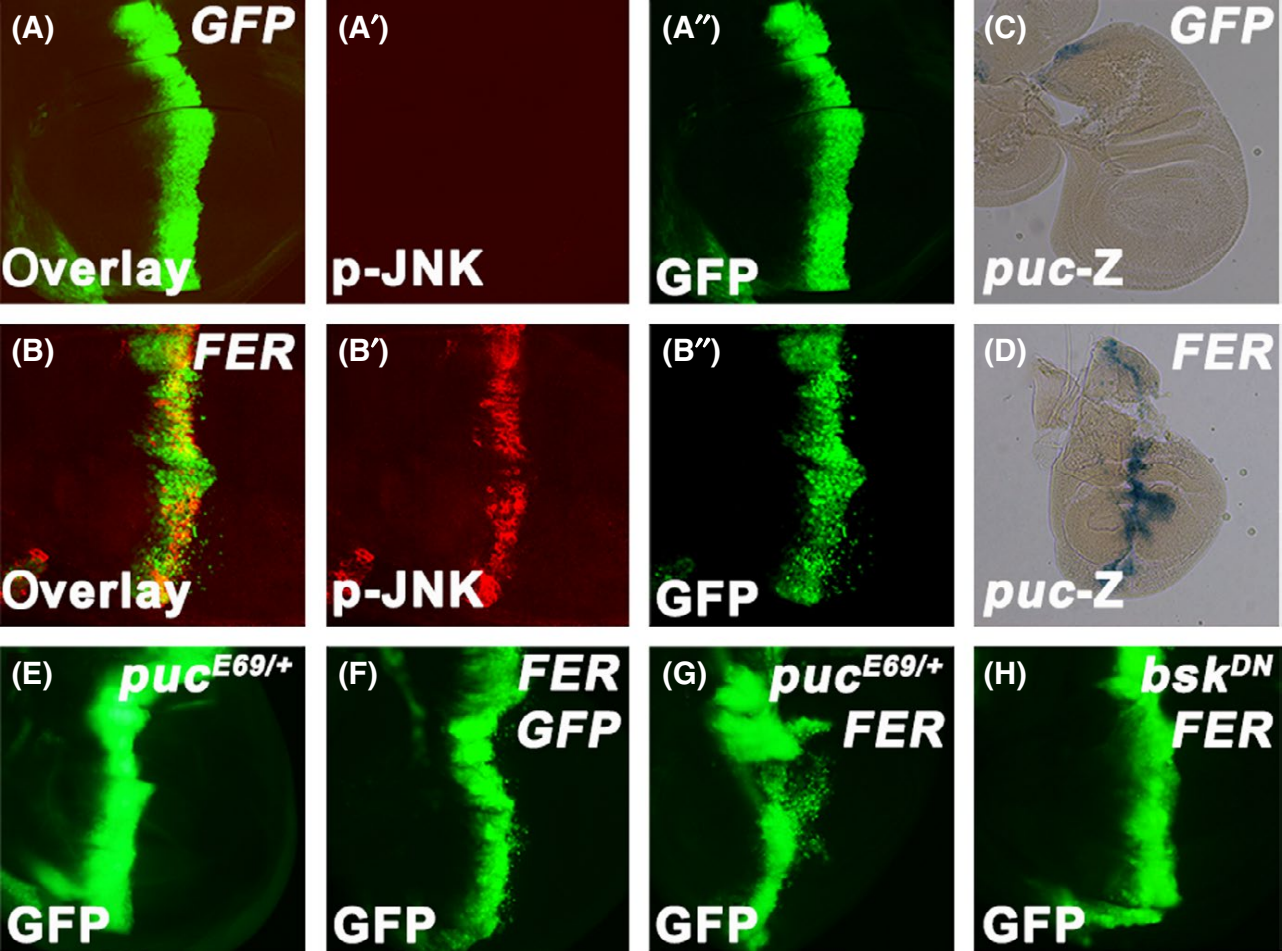
FER activates JNK signalling. A and B, Immunostaining of p‐JNK in the wing discs expressing *ptc‐Gal4 UAS‐GFP*/*+*; *UAS‐GFP*/*+* (A), *ptc‐Gal4 UAS‐GFP*/*+*; *UAS‐FER*/*+* (B). C and D, X‐Gal staining of a *puc‐Z* reporter gene in the wing discs expressing *ptc‐Gal4 UAS‐GFP*/*+*; *UAS‐GFP*/*puc^E69^* (C), *ptc‐Gal4 UAS‐GFP*/*+*; *UAS‐FER*/*puc^E69^* (D). E‐H, The migration of the GFP‐positive cells at the A/P compartment boundary of the wing discs expressing *ptc‐Gal4 UAS‐GFP*/*+*; *UAS‐GFP*/*puc^E69^* (E), *ptc‐Gal4 UAS‐GFP*/+; *UAS‐FER*/ *UAS‐GFP* (F), *ptc‐Gal4 UAS‐GFP*/*UAS‐GFP*; *UAS‐FER*/*puc^E69^* (G), *ptc‐Gal4 UAS‐GFP*/*+*; *UAS‐FER*/*UAS‐bsk^DN^* (H)

Next, we investigated how FER induced cell migration. Activation of the JNK signalling pathway has been demonstrated to trigger cell migration and activate *MMP1* expression.^6^ Notably, overexpression of FER resulted in similar observations, which prompted us to examine whether the JNK pathway was involved in FER‐induced cell migration in the wing disc. We utilized anti‐phospho‐JNK (p‐JNK) antibody, which recognized the activated form of JNK, to directly monitor the JNK activity. In *ptc‐Gal4 > GFP* controls, the basal level of p‐JNK was detected throughout the disc, implying a requirement of the JNK signalling pathway for the development of normal wing disc. Whereas in *ptc‐Gal4 > FER* discs, aberrantly robust activation of JNK was observed in the FER‐expressing domain (Figure 2A‐2B). Besides, we further assessed the JNK activity using a *lacZ* reporter gene (*puc^E69^*, referred to as *puc‐*Z) in FER‐expressing lines. Consistent with the p‐JNK immunofluorescence, the *puc‐*Z expression was strongly elevated in the A/P boundary of the *ptc‐Gal4 > FER* wing disc (Figure 2C‐D).

As overexpression of FER activated the JNK signalling pathway, we continued to assess whether JNK signalling was required for FER‐induced cell migration. To test this notion, we altered the activity of JNK signalling in the *ptc*‐*Gal4* > *FER* background. To slightly elevate the JNK activity, we removed one copy of JNK negative regulator *puc* (*puc^E69/+^*). *puc^E69/+^* mutant alone could not affect the expression pattern of *ptc‐Gal4* in the wing discs (Figure 2E). However, this genetic alteration significantly aggravated the spreading of *ptc‐Gal4 > FER* cells (Figure 2F‐2G, and Figure S4). We also reduced the JNK signalling by expressing *bsk^DN^*, a dominant negative *bsk* construct. Co‐expression of *bsk^DN^* strongly suppressed the migration of *ptc‐Gal4 > FER* cells (Figure 2H and Figure S4). These results indicate that FER‐induced cell migration in the wing discs is mainly attributed to the activation of the JNK signalling pathway.

### 3.3 FER is required for JNK signalling

**Figure 3:**
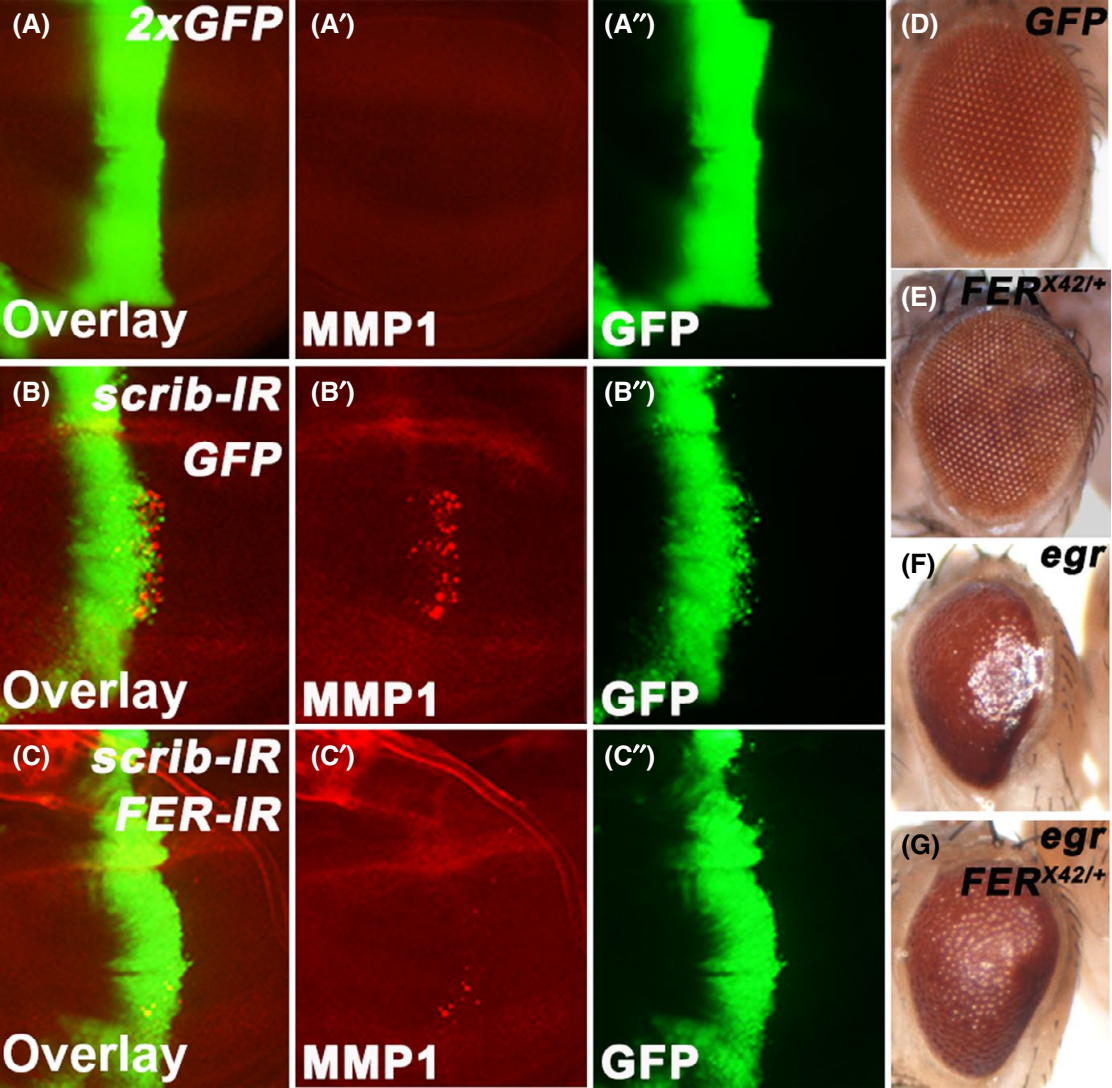
FER is required for JNK signalling. A‐C, Immunostaining of MMP1 in the wing discs expressing *ptc‐Gal4 UAS‐GFP*/*+*; *UAS‐GFP*/*UAS‐GFP* (A), *ptc‐Gal4 UAS‐GFP*/*UAS‐scrib‐IR*; *UAS‐GFP*/*+* (B), *ptc‐Gal4 UAS‐GFP*/*UAS‐scrib‐IR UAS‐FER‐IR* (C). (D‐G) Eye morphologies of flies expressing *GMR‐Gal4*/*+*; *UAS‐GFP*/*+* (D), *FER^X42^/+* (E), *GMR‐Gal4*/*+*; *UAS‐egr*/*+* (F) *GMR‐Gal4*/*+*; *UAS‐egr*/*FER^X42^* (G)

The above results suggested that FER was sufficient to activate JNK signalling. We further asked whether FER was necessary for the signal transduction of JNK signalling. Previous studies showed that the downregulation of cell polarity genes such as *dlg* or *scrib* resulted in JNK‐dependent cell migration.^36^, ^37^ Consistently, we observed that in *ptc*‐Gal4 > *scrib‐IR* discs, numerous *scrib* knockdown cells migrated away from A/P boundary, along with increased expression of *MMP1* (Figure 3A‐B). Importantly, we found that the knockdown of *FER* notably suppressed both phenotypes (Figure 3A‐C). In *Drosophila*, aberrant JNK activation not only induces cancer‐like phenotype but also affects biological process such as organ development. For example, ectopic expression of *eiger* (*egr*, the *Drosophila* tumour necrosis factor superfamily ligand) in the eyes driven by *GMR‐Gal4* activates the JNK signalling pathway.^38^, ^39^ This genetic manipulation triggers cell death during eye development and results in a small eye phenotype in adults. Several studies have reported that this phenotype can be rescued by reducing JNK activity.^38^, ^39^ We found that removing one copy of *FER* or knocking down *FER* largely alleviated *egr*‐induced small eye phenotype (Figure 3D‐G, and Figure S5). Together, these results suggest that FER is required for the outputs of the JNK signalling pathway.

### 3.4 FER regulates JNK signalling through Bsk

**Figure 4:**
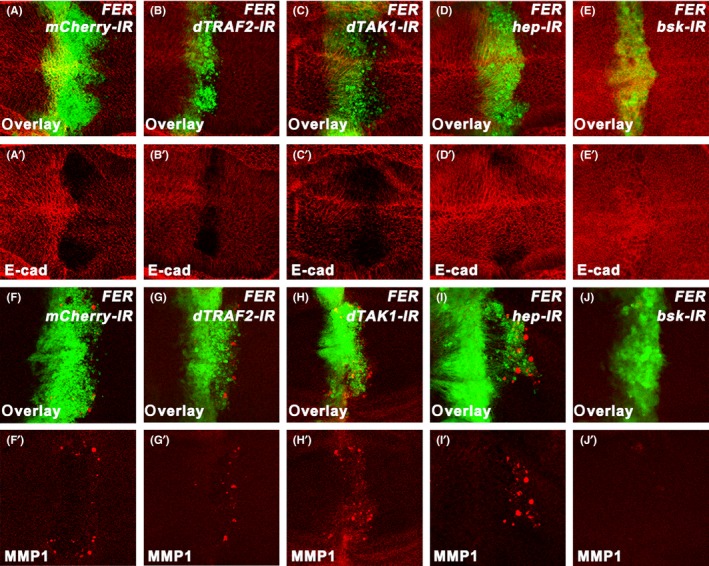
Genetic interaction between FER and JNK signalling. A‐J, Immunostaining of E‐cad (A‐E) and MMP1 (F‐J) in the wing discs expressing *ptc‐Gal4 UAS‐GFP*/*+*; *UAS‐FER*/*UAS‐mCherry‐IR* (A and F), *ptc‐Gal4 UAS‐GFP*/*+*; *UAS‐FER*/*UAS‐dTRAF2‐IR* (B and G), *ptc‐Gal4 UAS‐GFP*/*+*; *UAS‐FER*/ *UAS‐dTAK1‐IR* (C and H), *ptc‐Gal4 UAS‐GFP*/*+*; *UAS‐FER*/ *UAS‐hep‐IR* (D and I), *ptc‐Gal4 UAS‐GFP*/*+*; *UAS‐FER*/ *UAS‐bsk‐IR* (E and J)

**Figure 5:**
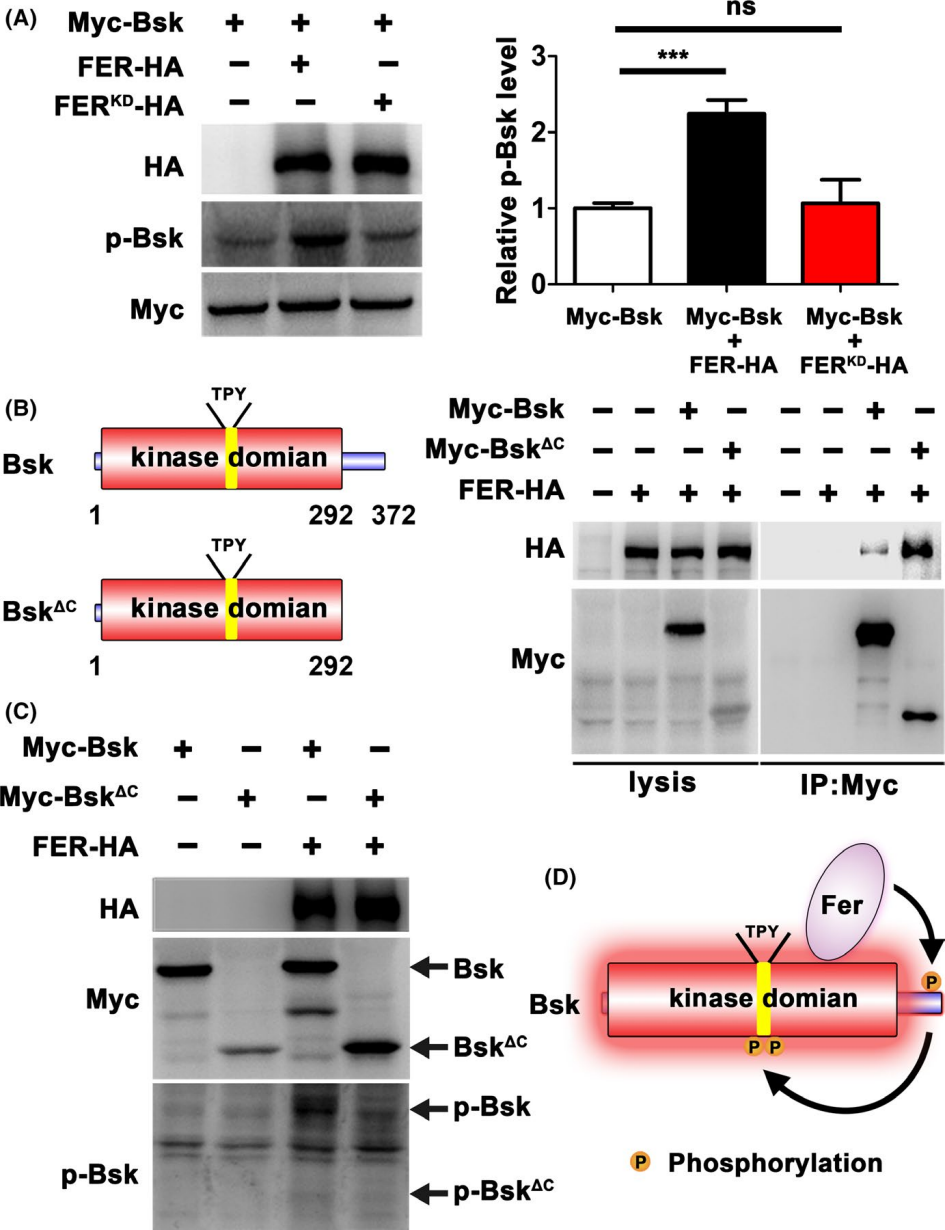
FER activates JNK signalling through Bsk. A, The Bsk phosphorylation levels in the absence and presences of FER or FER^KD^. The relative p‐Bsk levels were normalized to the Myc‐Bsk levels. B, The interaction between FER and two forms of Bsk. C, The phosphorylation levels of Bsk and Bsk^ΔC ^in the absence and presence of FER. D, The proposed model for the activation of Bsk by FER. All experiments are performed on *Drosophila* S2 cells. ***P < 0.001; ns, not significant. Data are presented as mean ± SEM (n = 3)

The above results indicated that FER was a positive regulator of the JNK signalling pathway. To genetically map the location of FER in this pathway, we disrupted JNK signal transduction by knocking down its key components in the *ptc*‐Gal4 > *FER* background. The JNK signalling pathway is transmitted by a series of precisely regulated kinases including Egr, dTRAF2, dTAK1, Hep and Bsk. Ectopic FER expression with *ptc*‐Gal4 (*ptc*‐Gal4 > *FER*) induced cell migration, accompanied by increased MMP1 and decreased E‐cad expression. We found that all of these phenotypes remained unaffected by knocking down *dTRAF2, dTAK1* or *hep*, but were strongly suppressed by knocking down *bsk* (Figure 4), indicating that *FER* might act downstream of or in parallel with *hep*, but upstream of *bsk*. Consistently, increased phosphorylation of Bsk was observed by co‐expressing FER in S2 cells (Figure 5A). In contrast, the p‐Bsk levels remained unaffected by the co‐expression of the kinase‐dead FER^K1092M^ (FER^KD^) mutant (Figure 5A), suggesting that the kinase activity of FER is required for its function in Bsk. We performed the co‐immunoprecipitation (coIP) assay and found that FER interacted with Bsk (Figure 5B). Bsk consists of an N‐terminal protein kinase domain (amino acids [aa] 1‐292) and a short C‐terminal extension (aa 293‐372), and the anti‐p‐JNK antibody specifically detect the phosphorylation events in the TPY motif of the kinase domain.^40^ To determine which region of Bsk mediates its interaction with FER, we constructed Bsk^ΔC ^(aa 1‐292), which lacked the C‐terminal region. Unexpectedly, the interaction between FER and Bsk^ΔC^ was much stronger than that between FER and the full‐length Bsk (Figure 5B). Typically, phosphorylation of a substrate significantly reduces its affinity to its kinase.^41^, ^42^ The above data thus suggest that FER binds to Bsk at the kinase domain but phosphorylates Bsk at its C‐terminus. The lack of the phosphorylation site in Bsk^ΔC^ stabilized FER‐Bsk interaction. In consistent with this hypothesis, Bsk^ΔC^ was not activated by FER (Figure 5C), indicating that the phosphorylation at the C‐terminus by FER primes for Bsk activation. Taken together, we propose a molecular mechanism by which FER activates Bsk (Figure 5D). FER binds to the kinase domain of Bsk and phosphorylates its C‐terminus, which consequently triggers Bsk activation in the TPY motif.

### 3.5 Fer regulates the JNK signalling pathway in mammalian cells

**Figure 6:**
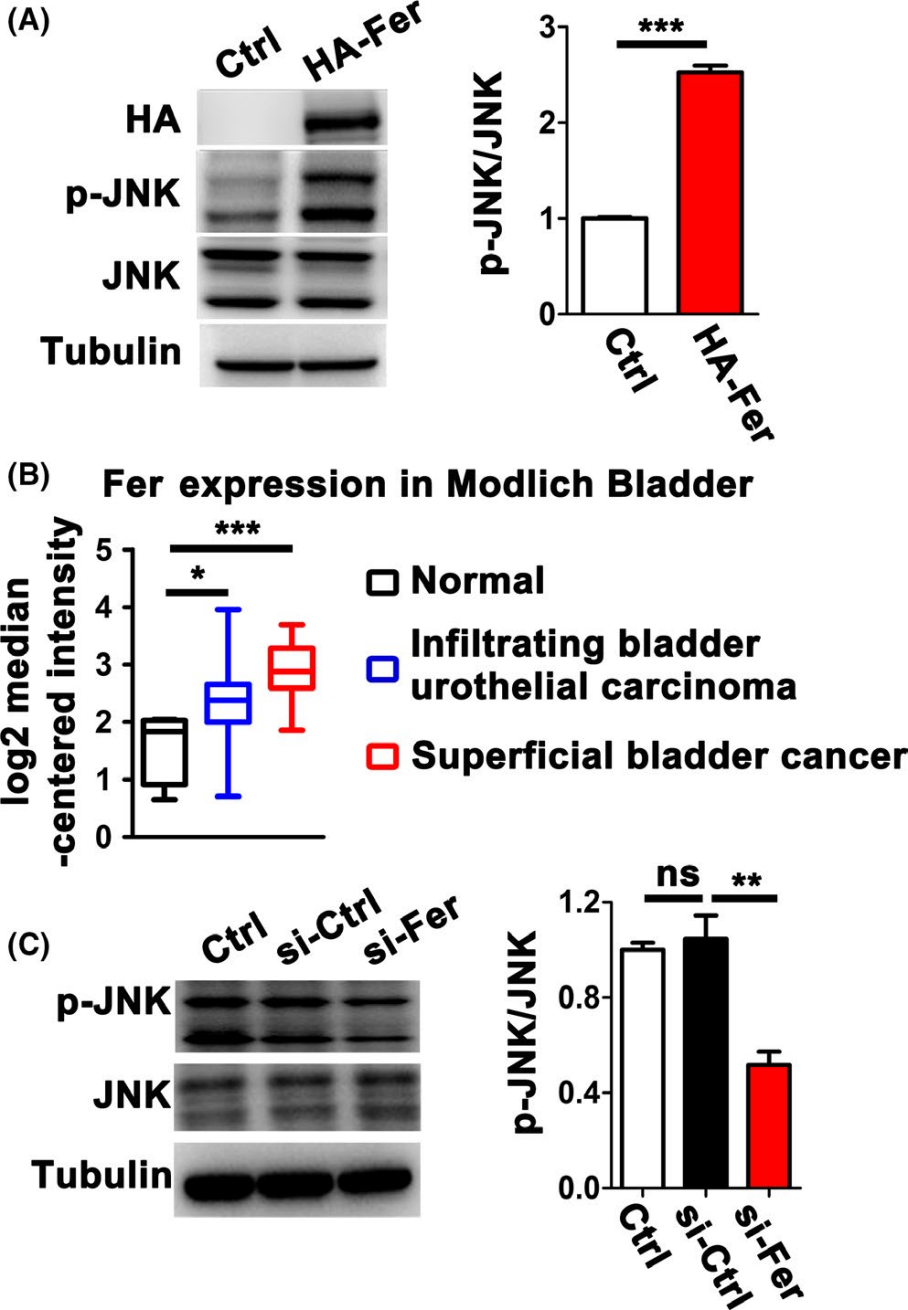
FER regulates JNK signalling in mammalian cells. A, The JNK phosphorylation levels in the absence and presences of Fer in 293T cells. B, Fer expression in Modlich Bladder. Dataset is grouped by cancer type. The reporter used in the study to detect Fer expression is J03358. C, The phosphorylation levels of JNK in T24 cells with or without Fer knockdown. *P < 0.05; **P < 0.01; ***P < 0.001; ns, not significant. Data are presented as mean ± SEM (n = 3)

Having demonstrated that Fer promotes cell migration through activating the JNK signalling pathway in *Drosophila*, we next checked whether Fer played a conserved role in mammals. In 293T human embryonic kidney cells, overexpression of Fer largely increased the endogenous p‐JNK levels (Figure 6A). Fer overexpression is also observed in the pathological context. For example, Oncomine analysis (https://www.oncomine.org) showed that Fer was highly amplified in metastasized bladder cancers (Figure 6B). T24 is a well‐characterized human urinary bladder carcinoma cell line with high expression of Fer. To test the idea whether upregulated Fer participated in the regulation of the JNK signalling pathway in cancer cells, we knocked down Fer in T24 cells with Fer siRNA (si‐Fer; Figure S6) and observed that *Fer* RNAi significantly decreased the activity of endogenous JNK (Figure 6C). Together, these results suggest that Fer regulates the JNK signalling pathway in mammals.

## 4 DISCUSSION

Although aberrant activation or overexpression of Fer is found in various cancers,^20^, ^22^ the pathological consequence and the underlying mechanism of this misregulation remain elusive. In this study, we showed that overexpression of FER in *Drosophila* wing disc resulted in cell migration, a key step in the progression of cancer (Figure 1). Moreover, we observed that in *scrib*‐IR‐induced cell migration model, the knockdown of FER strongly suppressed the migration phenotype (Figure 3A‐C), which further validated the role of FER in regulating cell migration.

Given the pivotal role of FER in regulating cell adhesion,^19^ the migrated behaviour in FER overexpressed cells could either be a secondary effect of the defect in cell adhesion or the consequence of cell migration. In this study, we ascribed it to the later for two reasons. Cells with disrupted adhesion often disperse into the surrounding wild‐type cells without a direction.^43^ However, FER‐induced cell migration is unidirectional. We noted that *ptc*‐Gal4 > *FER* cells migrated from the A/P boundary to the posterior compartment of discs (Figure 1A‐B). In addition, ectopic expression of FER caused aberrant MMP1 expression (Figure 1C‐D), which is a characteristic for cell migration.^6^

MMP1 is not only the hallmark of cell migration but also a target gene of JNK signalling.^6^ Previous studies have also shown that JNK signalling is closely associated with cell migration. Besides, it has been reported that gain‐of‐function FER mutant *Drosophila* displays dorsal closure defect and axon misrouting. Co‐expressing *puc* is able to substantially correcting both of these phenotypes.^44^ We thus analysed the role of the JNK signalling pathway in FER‐induced cell migration in the wing discs. We found that JNK signalling was remarkably activated by overexpressing FER (Figure 2A‐2D). More importantly, elevating JNK activity by deleting one copy of *puc* aggravated FER‐induced cell migration. Conversely, reducing JNK activity by expressing dominant negative *bsk* suppressed this phenotype (Figure 2E‐H). Taken together, we demonstrate that JNK signalling is responsible for FER‐induced cell migration. As Fer has been shown to be involved in tumour metastasis in human cancers, it will be interesting to investigate whether JNK signalling accounts for Fer‐mediated metastasis in mammals.

The JNK signalling pathway is tightly regulated and is implicated in a plethora of cellular function.^45^, ^46^ However, the molecular mechanism that modulates the JNK signalling pathway remains elusive. Here, we reported that FER was able to positively regulate JNK signalling, which was supported by several lines of evidence. First, as mentioned above, overexpression of FER was sufficient to activate the JNK signalling pathway (Figure 2A‐D). Second, FER was necessary for the outputs of the JNK signalling pathway. Knocking down *FER* alleviated JNK‐dependent invasion‐like phenotype in the wing discs (Figure 3A‐C). *FER* RNAi or *FER* mutant also rescued JNK‐induced small eye phenotype (Figure 3D‐G, and Figure S5). Third, FER interacted with and promoted the activity of Bsk (the *Drosophila* JNK; Figure 5). Last, Fer regulated JNK activities in mammalian cells (Figure 6). It has been reported that Fer is activated in response to reactive oxygen species (ROS) in a variety of cell types.^47^ In addition, ROS are important factors in the activation of the JNK signalling pathway. There is a possibility that ROS may regulate Fer‐induced JNK activation.

In conclusion, our work provides new insights into the roles of FER in regulating cell migration and JNK signalling and reveals a potential novel therapeutic target for cancer metastasis.

## CONFLICT OF INTEREST

The authors declare no conflict of interest.

## AUTHOR CONTRIBUTIONS

HS designed the research; PL, ZM, Y.Y and XH performed the experiments; HS and PL analysed the results and wrote the paper.

## Data Availability

The data that support the findings of this study are available from the corresponding author upon reasonable request.
