# Supplementary material for: FER promotes cell migration via regulating JNK activity
Source: Cell Prolif. 2019 Jul 1;52(5):e12656. doi: 10.1111/cpr.12656 (PMC6797522; doi:10.1111/cpr.12656)
Supplement: Supplementary file 1 [file CPR-52-e12656-s001.doc]

**Supplementary Information**

**
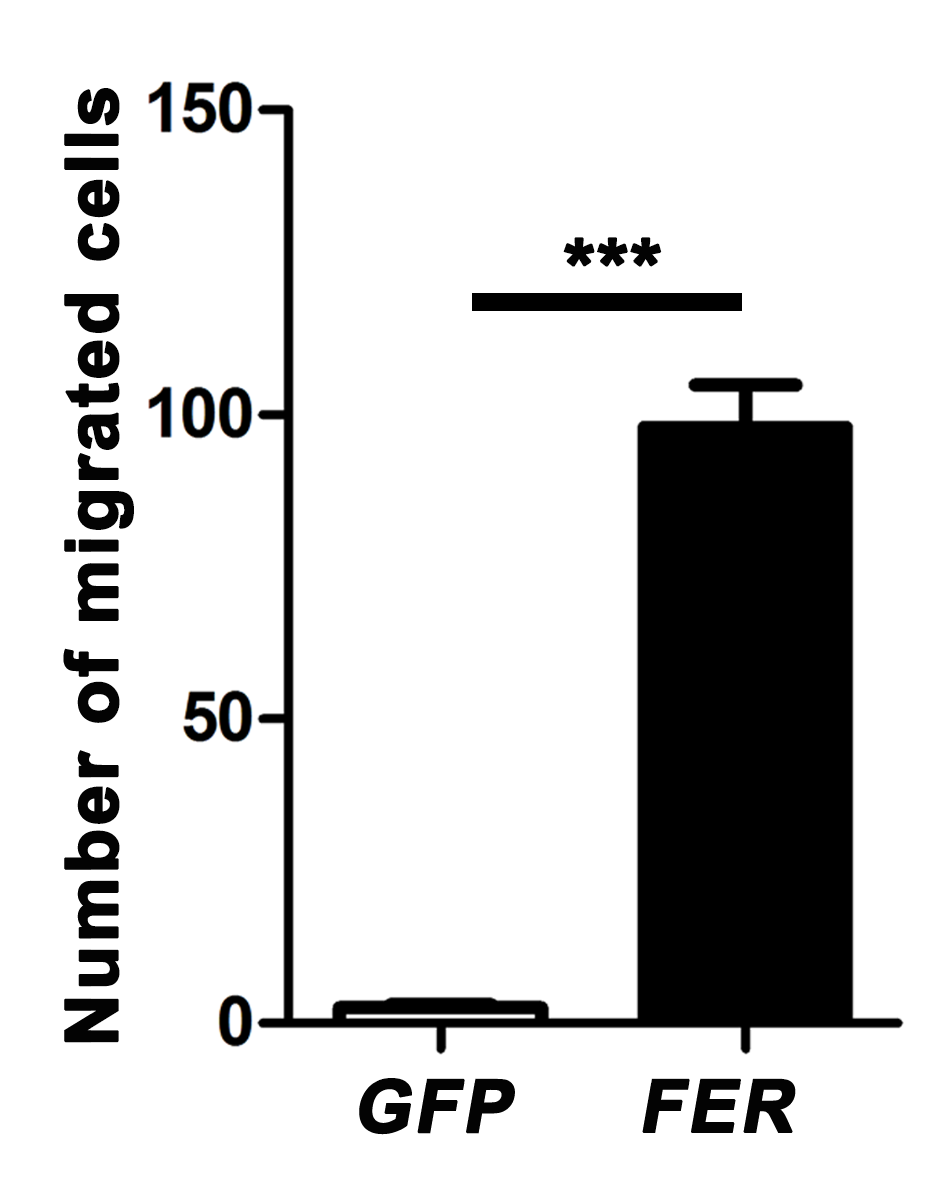
**

**Figure S1 Overexpression of FER induces cell migration.** Quantification of the migrated cells in Figure 1A-1B. ***p<0.001. Data are presented as mean±SEM (n=10).


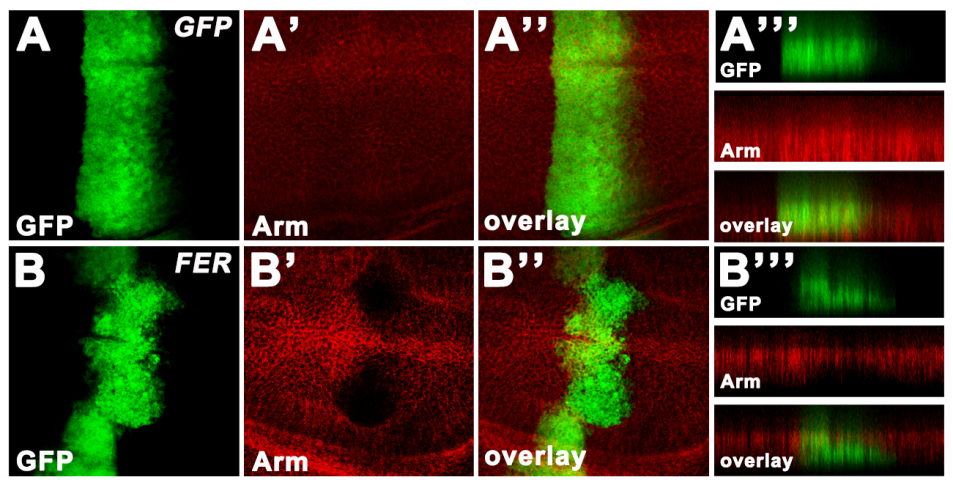


**Figure S2** **FER overexpression leads to down-regulation of Arm.** (A) Immunotaining of Arm in the wing discs expessing *ptc-Gal4 UAS-GFP*/*+*; *UAS-GFP*/*+*. (A’’’) Z stack images in A-A’’. (B) Immunotaining of Arm in the wing discs expessing *ptc-Gal4 UAS-GFP*/*+*; *UAS-FER*/*+*. (B’’’) Z stack images in B-B’’.


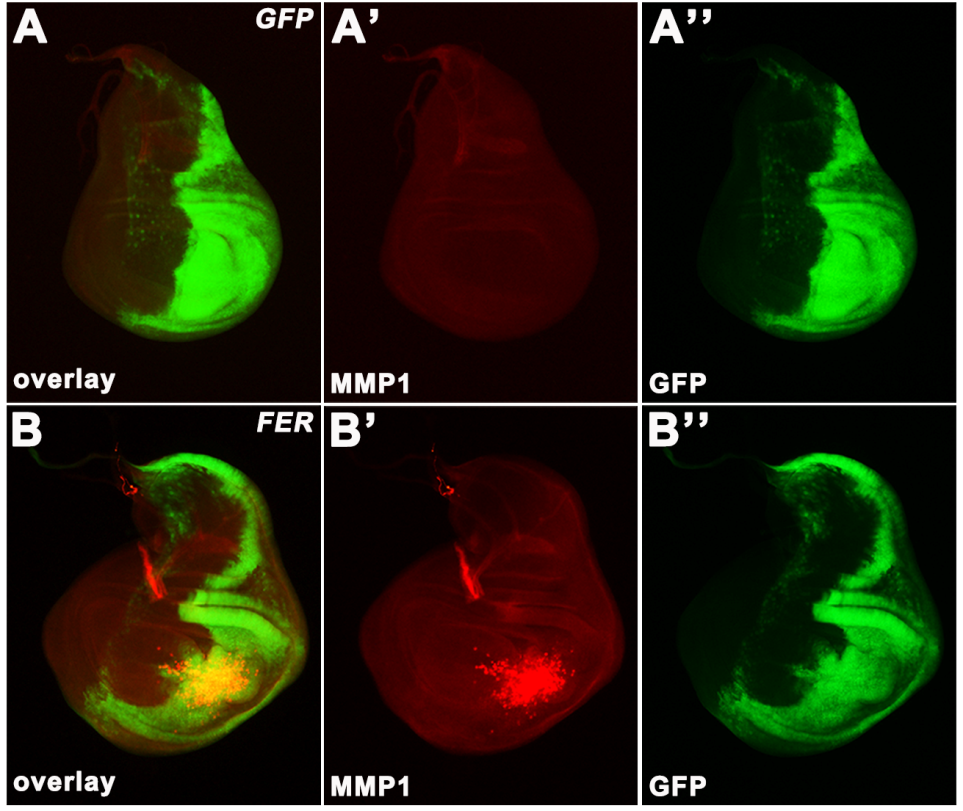


**Figure S3 FER overexpression results in up-regulation of MMP1.** (A) Immunotaining of MMP1 in the wing discs expessing *en-Gal4 UAS-GFP*/*+*; *UAS-GFP*/*+*. (B) Immunotaining of MMP1 in the wing discs expessing *en-Gal4 UAS-GFP*/*+*; *UAS-FER*/*+*.


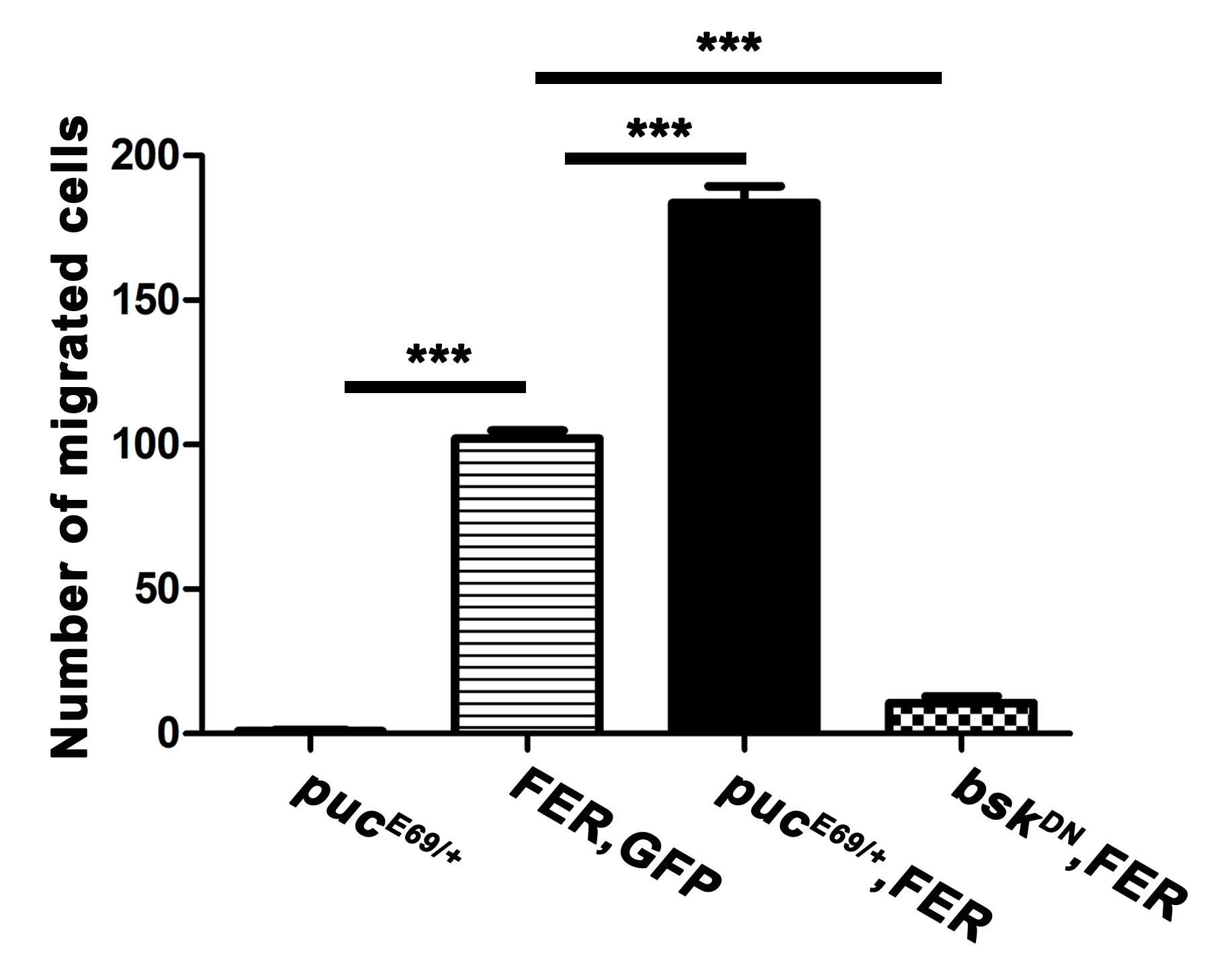


**Figure S4 The effects of alterating JNK signaling activity on FER-induced cell migration.** Quantification of the migrated cells in Figure 2E-2H. ***p<0.001. Data are presented as mean±SEM (n=10).


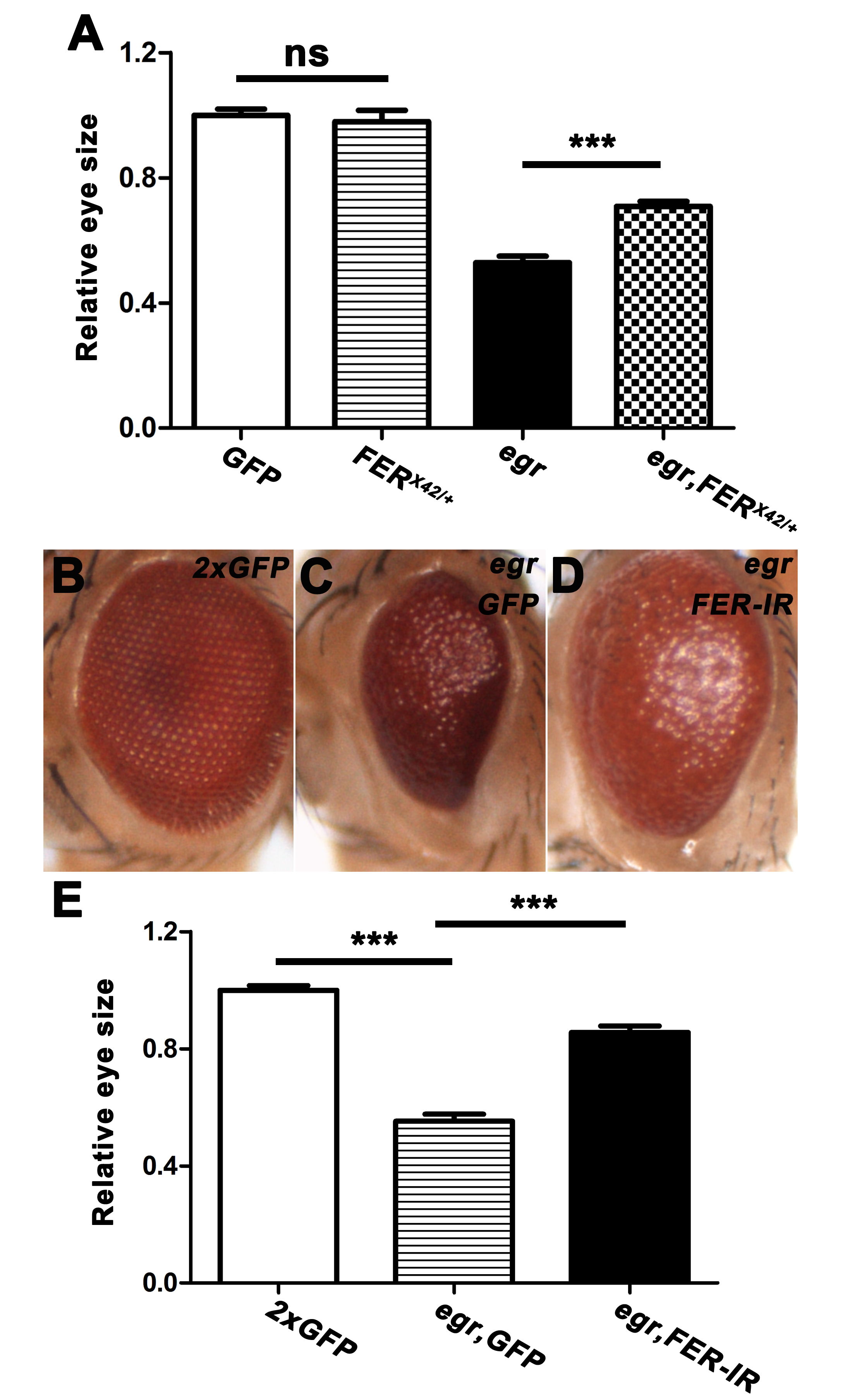


**Figure S5 FER is required for JNK-dependent eye morphology.** (A) Quantification of the eye sizes in Figure 3D-3G. (B) Eye morphologies of flies expressing *GMR-Gal4*/*+*; *UAS-GFP*/*UAS-GFP*. (C) Eye morphologies of flies expressing *GMR-Gal4*/*+*; *UAS-egr*/*UAS-GFP.* (D) Eye morphologies of flies expressing *GMR-Gal4*/*+*; *UAS-egr*/ *UAS-FER-IR*. (E) Quantification of the eye sizes in (B-D). ***p<0.001; ns, not significant. Data are presented as mean±SEM (n=10).


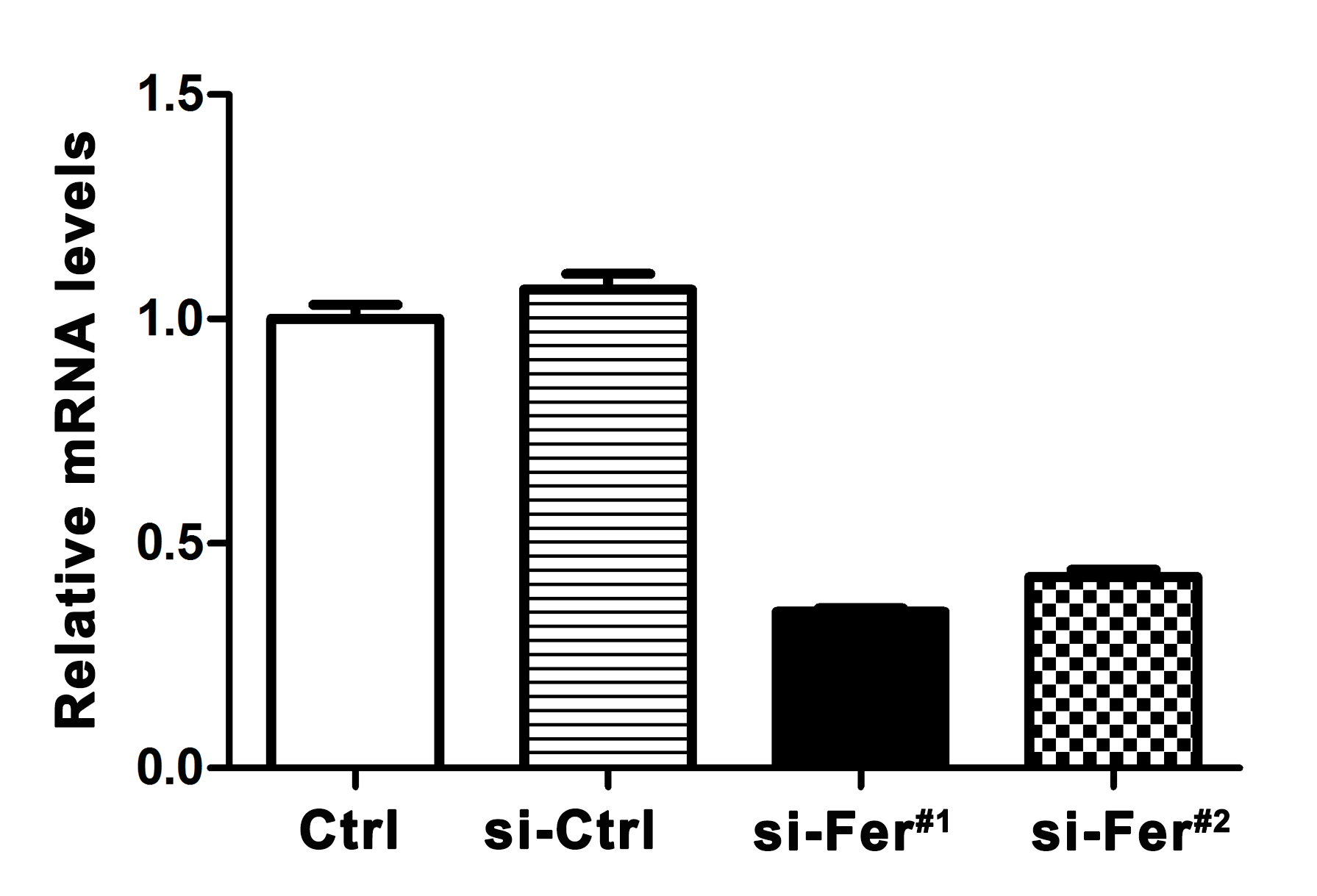


**Figure S6 The knockdown efficiency of Fer.** Relative Fer mRNA levels in T24 cells with or without Fer knockdown.
